# Why do physicians lack engagement with smoking cessation treatment in their COPD patients? A multinational qualitative study

**DOI:** 10.1038/s41533-017-0038-6

**Published:** 2017-06-23

**Authors:** Eva Anne Marije van Eerd, Mette Bech Risør, Mark Spigt, Maciek Godycki-Cwirko, Elena Andreeva, Nick Francis, Anja Wollny, Hasse Melbye, Onno van Schayck, Daniel Kotz

**Affiliations:** 1 Department of Family Medicine, Maastricht University Medical Centre, CAPHRI School for Public Health and Primary Care, Maastricht, The Netherlands; 2 Department of Community Medicine, General Practice Research Unit, UiT The Arctic University of Tromsø, Tromsø, Norway; 3 Centre for Family and Community Medicine, Division of Public Health, Faculty of Health Sciences, Medical University of Lodz, Lodz, Poland; 4 Department of Family Medicine, Northern State Medical University, Arkhangelsk, Russia; 5 Division of Population Medicine, Institute of Primary Care and Public Health, Cardiff University, Cardiff, UK; 6 Department of General Practice, University Medical Centre, Rostock, Germany; 7 Institute of General Practice, Addiction Research and Clinical Epidemiology Unit, Heinrich-Heine-University Düsseldorf, Düsseldorf, Germany

## Abstract

Smoking cessation is the only effective intervention to slow down the accelerated decline in lung function in smokers with chronic obstructive pulmonary disease. Nevertheless, physicians often do not routinely provide evidence-based smoking cessation treatment to their patients. To understand underlying reasons, we explored how physicians engage in smoking cessation treatment in their chronic obstructive pulmonary disease patients. In total, 21 focus group discussions were held with general practitioners and pulmonologists in seven different countries in Europe and Asia. We generated three themes, whereby some of the issues concerned smokers in general: first, ‘physicians’ frustration with chronic obstructive pulmonary disease patients who smoke’. These frustrations interfered with the provision of evidence-based treatment and could result in this group of patients being treated unequally. Second: ‘physicians’ limited knowledge of, and negative beliefs about, smoking cessation treatment’. This hindered treating smokers effectively. Third: ‘healthcare organisational factors that influence the use of smoking cessation treatments’. Money and time issues, as well as the failure to regard smoking as a disease, influenced how physicians engaged in smoking cessation treatment. Our results indicate that there is a number of barriers to the provision of effective smoking cessation treatment in patients with chronic obstructive pulmonary disease and smokers in general. Introducing an informative smoking cessation programme, including communication skills and ethical issues, in the vocational and postgraduate medical training may help to address these barriers. This is important in order to increase engagement with smoking cessation treatment and to improve quality of chronic obstructive pulmonary disease care.

## Chronic lung disease: Changing attitudes to help patients stop smoking

Doctors should be given careful, ethically-informed guidance during medical training to help them to support patients to quit smoking. The most important part of treatment for patients with chronic obstructive pulmonary disease (COPD) is help to stop smoking. However, there is evidence to suggest that doctors don’t always motivate COPD patients to quit. Eva Anne Marije van Eerd at Maastrict University, The Netherlands, together with an international team of scientists, conducted focus group interviews with doctors in seven different countries to assess barriers to smoking cessation. They found that doctors’ frustration with and negative attitudes towards patients who continued to smoke contributed to poor cessation management and treatment inequalities in some cases. Many doctors also cited a lack of experience with smoking cessation techniques alongside time and money issues as barriers to effective treatment.

## Introduction

Chronic obstructive pulmonary disease (COPD) is a major public and individual health problem. Experts think that, by 2020, COPD will rank fifth worldwide in terms of burden of disease and third in terms of mortality.^1^ Higher mortality rates and an accelerated decline in lung function are seen in patients with COPD who continue smoking.^1^ Smoking cessation is therefore the most important treatment for patients with COPD, as it is the only evidence-based intervention which has been proven to slow down the accelerated decline in lung function.^1^

To promote smoking cessation, international COPD guidelines include recommendations for effective smoking cessation interventions.^1, 2^ They state, in accordance with the Cochrane review on smoking cessation in patients with COPD, that a combination of behavioural and pharmacological interventions is superior to any other treatment.^3^ Nevertheless, the prevalence of smoking in patients with COPD is still high and there is evidence that it exceeds the rate of smoking in the general population.^4^ There is only limited evidence that patients get motivated to quit on receiving a diagnosis of COPD.^5^ This implies that many COPD patients still continue smoking even though quitting is their best treatment option.

Physicians play a very important role in providing effective smoking cessation treatment for these patients. However, literature shows that they do not routinely deal with smoking cessation during their consultations with smokers.^6–8^ According to a large European review, several factors seem to influence physicians’ engagement in smoking cessation: patient characteristics; physician-characteristics, including attitude and cessation-specific knowledge and skills; structural factors, including reimbursement and time required.^8^ A different review found that some physicians had negative thoughts about smoking cessation treatment as they considered it time-consuming and ineffective, and they lacked confidence in their ability to discuss smoking cessation with their patients.^9^

To our knowledge, no attention has been given in the literature specifically to physicians’ views on smoking cessation treatment for smokers with COPD. Whereas, for this group of smokers, it is more urgent that they quit smoking, while it is even more difficult due to their higher addiction levels and higher susceptibility to developing depressive symptoms.^10–12^ There are studies exploring experiences with COPD care in general, but these studies do not specifically address smoking cessation.^13–16^ From these studies, it is apparent that engagement in COPD care is influenced by physician-related factors such as perceived low self-efficacy in the provision of treatment and lack of confidence in the effects of treatments.^13, 16^ Also, patient-related factors such as being non-compliant and not taking responsibility for their condition were barriers for carrying out COPD care.^15^ Furthermore, awareness of available treatments and support for patients with COPD, as well as the time required for such treatments seemed to create organisational barriers.^14^ It is not clear whether these factors also hinder smoking cessation care in patients with COPD or if there are different issues in this specific population.

The aim of this study was therefore to explore how physicians engage in smoking cessation treatment for their COPD patients, by exploring their attitudes, reported practice and experienced problems in this regard. Once researchers and physicians understand specific needs and concerns regarding utilising cessation treatments for this particular group of smokers, they may address these problems in order to increase smoking cessation in COPD patients.

## Results

### Physicians’ frustration with COPD patients who smoke

Many physicians shared negative feelings towards patients with COPD who continued smoking. This seems to hinder them in providing evidence-based treatment, including smoking cessation treatment.

Physicians seemed to feel responsible for motivating patients to quit smoking.

‘Yes, make them, make them to wish to stop smoking (laughs a bit) is what we should do.’ [FGD 1, Norway, GP]

Several discussions focused on how exhausting it is for physicians to spend their valuable time on COPD patients and to prescribe expensive medication to relieve symptoms. Nevertheless, many physicians seemed to conflate the importance of supporting patients to quit smoking with a personal desire to try and make them stop. They reported frustration regarding patients who demanded treatment but refused to accept responsibility for the treatment themselves.

‘They [COPD patients that smoke] are a very difficult patient group, especially those that are just on the cusp of needing long term oxygen and you’re trying to convince them that you’re not going to give it to them if they don’t stop smoking … it’s very frustrating if they still persist…’ [FGD 3, Wales, Pulmonologist]

Other frustrations that hindered physicians in working with smokers with COPD were difficulties in communication. Physicians reported that some patients no longer attended surgery because they do not like hearing about smoking cessation or feel guilty about their smoking behaviour.

‘It’s difficult to treat and handle this group of patients, right? You need to strongly motivate them in order to [make them stop smoking] … if you press them too much you will reject them; if you press them too hard to make them stop smoking you will make them your enemies: that’s the fine line.’ [FGD 1, Norway, GP]

Some also believed patients were ‘lying’ about their smoking behaviour. Others reported that COPD patients, when questioned about their smoking behaviour, consciously or unconsciously, trivialized their smoking behaviour.

‘I switched to ‘light’ cigarettes’ and, ‘Doctor, I’ve quit! I have not smoked for three days!’ He smoked for 50 years before’. [FGD 2, Russia, pulmonologists]

Eventually, all these frustrations resulted in a general feeling of dissatisfaction with regards to supporting COPD patients with quitting smoking.

‘…it is almost like tilting against windmills.’ [FGD 3, Poland, pulmonologist]

These negative experiences with smokers with COPD seemed to contribute to physicians being prejudiced against smokers with COPD. Some physicians discussed the issue that these prejudgements provoke the risk of treating smokers with COPD differently. Some said that they found smokers with COPD ‘nonsensical’ and it was their own fault they had COPD, and were more reluctant to prescribe expensive medication. Another physician said that if a COPD patient had quit smoking ‘he has a plus in our view and we should take care of him’ [FGD 1, Poland, GP].

In conclusion, these physicians felt responsible for ‘making’ COPD patients quit smoking. However, they experienced many patient-related barriers which frustrated them. These frustrations provoked the risk of developing negative feelings about this group of patients, and subsequently treating them unequally and potentially depriving them of the most effective treatments.

### Physicians’ limited knowledge of, and negative beliefs about, smoking cessation treatment

Many of the physicians felt they lacked experience and confidence in helping patients to stop smoking. Some also questioned the effectiveness of smoking cessation treatments. In the discussion of this, the physicians seemed inclined to switch from COPD-specific topics to topics regarding the general smoking population.

Even though all the physicians emphasised that smoking cessation is the most essential treatment for smokers with COPD, some of them questioned whether this also applied to particular groups of patients, e.g., late stages of COPD.

‘No, it has been proven that if you do not smoke you are better off; if you quit at a young age you are better off… but those people who are at a certain stage of COPD …those data are not convincing.’ [FGD 3, Netherlands, pulmonologist] (There are studies that show that also smokers in severe and very severe stages of COPD benefit from quitting smoking in terms of their quality of life and lung function^17, 18^)

Physicians from the Netherlands, Germany and Norway expressed the view that there were COPD patients who did not necessarily feel better after quitting smoking. It was difficult for them to explain the benefits of quitting to their patients without the prospect of a positive result.

Furthermore, several physicians stated that they were inexperienced in providing smoking cessation treatment. They reported finding it hard to talk to patients about smoking habits, regarding it as a delicate subject. They would also like to have tools for the promotion of smoking cessation, such as tools for communication and patient education. This was one of the topics of discussion about smoking cessation in general and not only about smokers with COPD.

‘We need resources on creating an atmosphere and educating patients…in order to increase their motivation to quit smoking and increase the success rate…’ [FGD 3, Hong Kong, GP]

Besides Hong-Kong, Norway and Russia in particular brought up this issue. In Russia, they also discussed the effectiveness of pharmacotherapy in the general smoking population. In Germany, there seemed to be a lack of trust in nicotine replacement therapy (NRT). Physicians in both Russia and Germany discussed the view that these aids would only delay quitting and not cure these patients (NRT has been proven to improve long-term abstinence from tobacco^19^). In addition, smoking cessation medication was said to be very expensive and therefore not widely used. In Germany, the physicians concluded that the most important element in smoking cessation is the communication with the patient.

‘And the communicative intervention, so to say, is more successful [in motivating quitting] than, for instance, a nicotine patch.’ [FGD 3, Germany, pulmonologist]

Besides these general discussions, there were some conversations that focussed more on smokers with COPD. The tenor of these discussions was speculative about communication, educational and social tools for motivating COPD patients to quit. For example, some physicians thought it might help to dramatise and scare the patient and they would like to have tools to illustrate the harm smoking does to your lungs (Confronting smokers with undetected COPD in a randomised controlled trial did not increase the rate of smoking cessation^20^; however, in a recent cohort study, a new diagnosis of COPD seemed to be linked to smoking cessation^5^). Furthermore, social influences were discussed as motivational tool: for example, a story was told about a grandfather who quit smoking when his granddaughter said he was not allowed to die yet.

### Healthcare organisational factors that influence the use of smoking cessation treatments

In this theme, the influence of health care organisational factors on smoking cessation treatment is discussed. In these discussions, the physicians broadened their views to include issues related to smoking cessation in general.

Smoking cessation treatment, in most countries, did not appear to be common practice in COPD care. Physicians from the Netherlands, Wales, Germany and Hong Kong reported lack of time and financial reimbursement as important obstacles for providing smoking cessation treatment.

‘It [quitting smoking] is part of the questionnaire, it is one of the items on the list you need to cover… but it is so time-consuming.’ [FGD 1, Netherlands, GP]

The physicians in the Netherlands felt there was not sufficient time in the COPD check-up appointment to discuss smoking cessation. In Germany, they grumbled about the price/quality ratio of the existing smoking cessation treatments.

‘It’s not economical but you shouldn’t lose your enthusiasm there.’ [FGD 3, Germany, pulmonologist]

The time spent on smoking cessation treatments is not recovered, as only a few people eventually quit smoking. In Russia and Germany, they discussed the importance of how smokers are ‘labelled’. This was not a COPD-specific discussion but concerned all smokers.

‘I consider that for some people, smoking is a disease, the same as alcohol dependency, for example.’ [FGD 3, Russia, GP]

In line with this, they discussed the availability and reimbursement of facilities for smoking cessation and compared it to facilities for other addictions in Germany.

‘Every alcoholic receives a detox, but a nicotine patient has to pay for his addiction himself. And this isn’t fair as both are partly recognised as addictions.’ [FGD 3, Germany, GP]

Apparently, in some countries, insurance companies and other important institutions in healthcare organisation consider smoking addiction to be different from other addictions. They do not provide the same facilities for smokers as for other addicted patients, such as alcoholics, whilst smoking is officially recognised as a drug-related illness by addiction treatment care.

## Discussion

### Main findings

In the focus group discussions (FGDs), the physicians mentioned patient-related, physician-related and healthcare organisational factors that hindered the routine provision of evidence-based smoking cessation treatment to patients with COPD. Some of the issues were not specific for smokers with COPD, but concerned smokers in general. Firstly, physicians experienced great frustration regarding smokers with COPD. In their view, smokers with COPD tended not to accept responsibility for their treatment; often were not honest about their smoking habits; trivialized the consequences of smoking and avoided health care visits. These frustrations provoked the risk of developing negative feelings about this group of patients, and treating them unequally by depriving them of effective treatments. Secondly, physicians lacked experience with different smoking cessation treatments. Thirdly, money and time issues, but also the failure to regard smoking as a disease, influenced physicians’ treatment of smokers with COPD and also smokers in general.

### Interpretation of findings in relation to previously published work

Physicians experienced frustration when working with smokers with COPD, as these patients were perceived as not taking responsibility for their disease. Present societal norms emphasise personal responsibility for one’s health^21^ and physicians expect their patients to act in the interest of successful health maintenance.^22^ Smokers with COPD fall short of these expectations as their disease has been caused by their own smoking behaviour, and many do not act to stop the progression of their disease by quitting. In this societal context, smokers with COPD feel stigmatised and they might be hesitant to contact their physicians because they fear criticism^21^ and become irritated by their advice to stop smoking.^23^ However, research shows the vast majority of smokers are happy for their physician to raise the topic of smoking, if the advice is given in an empathetic and patient-centred manner.^23–25^ In order to create an environment with less frustration, it is important that physicians contribute to reducing the negative effect of stigma,^21^ as this interferes with the physician-patient relationship. In our view, some physicians need to develop a less paternalistic style, and create room for the smokers’ own views.^12, 21^

Many physicians reported limited knowledge, confidence and skills regarding evidence-based smoking cessation treatments. A Europe-wide literature review demonstrated that the proportion of GPs that offer intensive smoking cessation interventions is low and that one of the reasons for this was limited knowledge and skills.^8^ This is confirmed by studies examining risk factor management and COPD management in general.^14, 26^ In national and international guidelines, there are overviews of the different effective smoking cessation options,^27, 28^ but literature has shown that physicians are not always familiar with these guidelines.^13^ Several factors can influence physicians’ adherence to guidelines, such as physicians’ view of their role in COPD care and how complex certain care is.^14^ In this study, we have seen that many physicians do not have faith in the guideline recommendations. Besides, the physicians mentioned that they thought communication was more important than prescribing pharmacotherapy and that it is difficult to familiarise oneself with communicative tools via theory. In order to increase skills and knowledge, it seems important to provide a smoking cessation programme in vocational training and postgraduate smoking cessation training for GPs. In this training, physicians should be taught evidence-based smoking cessation treatment and practise communication, besides reflection on personal norms and values.

Healthcare organisational factors influence how physicians engage in COPD care and smoking cessation. Each of the seven countries involved in this study organises COPD care in a different way. The Netherlands,^29^ Germany,^30^ Wales^31^ and Norway^32^ have the most structured COPD care. In Poland,^33^ Russia and Hong-Kong, the disease management programs seem to be less established. Even with structured care, however, there are several issues that complicate providing evidence-based smoking cessation treatment. Lack of time is often mentioned in the literature as a barrier to giving smoking cessation treatment.^8, 9^ In this study, the physicians further complained that the financial reward is insufficient. To our knowledge, there are no studies on the differences in outcomes when comparing the different healthcare systems. We can, however, see that in the Netherlands, Germany, Wales, Norway and Poland, the smoking rate is between 20–26%.^34^ In Russia, this number is much higher: 37.3% of people over 15 years of age smoke.^34^ In China, almost 50% of men smoke, but almost no women.^34^ It would be interesting to know if the way the healthcare system is organised influences smoking cessation, especially for smokers with COPD who, even at the best of times, experience difficulties in quitting.^35^

As smoking is officially recognised as an illness in addiction treatment care, it is interesting to note that in none of the countries rehabilitation programs for smoking are offered in the same way as rehabilitation from other drugs. This shows that there is still a stigma attached to smoking that considers it to be different from other addictions. If smoking is seen as an addiction such as with other drug addictions, then structured nicotine dishabituation programmes might be established and reimbursed.

### Strengths and limitations of this study

The primary dataset that we used was extensive, therefore the strength of this study is to be found in the participation of several countries in order to find common concerns regarding smoking cessation in different healthcare settings. We did not aim for a comparative study as a strict comparison between countries could not be made due to the plethora of information and scattered quotes on smoking. Furthermore, both GPs and pulmonologists attended the FGDs. This gave a fuller picture of physicians’ concerns regarding smoking cessation. In theme three, we briefly mention the importance of the different healthcare contexts for the interpretation and analysis of the information. A weakness in this study is that we were not able to specify exactly what role the different health contexts played for the construction of the concerns. Analysis of the data was done together with two researchers that participated in the primary data collection and analysis, and was proof read by all representatives from the different countries that participated. One other limitation is that the FGDs were originally designed to explore GPs’ and pulmonologists’ views and concerns on the management of COPD exacerbations in general. Smoking cessation is, of course, very important herein, but was not the main focus of the FGDs. On the other hand, this concomitantly resulted in important gut reactions on smoking related issues. This means that the results are reflections of most discussed concerns and we see them as hypothesis generating for future in-depth research in this field.

### Implications for future research, policy and practice

To promote physicians’ engagement in smoking cessation treatment, we advise introducing an informative smoking cessation programme, including communication and ethic, into the vocational training and postgraduate medical training. The attachment of stigma should be considered in this training and how this interferes with giving professional smoking cessation support. In addition evidence-based smoking cessation treatment should be taught and communication should be practiced. For future research, it would be interesting to take a more detailed look into the hypothesis generating results of this analysis including the influence of different healthcare systems on how smoking cessation treatment is performed. This will inform us about which elements are essential for performing effective smoking cessation treatment.

## Conclusions

Our results show physicians’ barriers to providing effective smoking cessation treatment in patients with COPD and smokers in general. This is influenced by general issues and attitudes regarding smoking cessation treatment and the management of patients with COPD. We saw a lot of frustration, inexperience and stigmatisation in physicians regarding smoking cessation in their COPD patients and in the general smoking population. Also, money and time issues hindered the physicians. It is important to increase engagement with smoking cessation treatment and thereby improve quality of COPD care.

## Methods

### Data collection

We used FGDs to explore how physicians comment on their engagement in smoking cessation treatment for COPD patients. A FGD design was found suitable as FGDs serve to create data within a group setting, and we wished to take advantage of the different inputs from physicians and their co-construction of meaning, rather than relying on individual interview data. Further, FGDs have the potential to bring forth and discuss different spontaneous viewpoints in relatively understudied domains, such as this domain.^36, 37^ We performed a secondary analysis together with researchers who had held the FGDs for the primary research. The FGDs were held in seven different countries in 2011–2012: Wales, the Netherlands, Poland, Russia, Germany, Norway and Hong Kong. These countries were selected due to earlier research collaboration on respiratory diseases. The FGDs were originally designed to explore general practitioners’ (GPs) and pulmonologists’ views and concerns on the management of COPD exacerbations in general.^15, 38^

Each country performed three FGDs with 4–7 participants considering that the chosen participants and the number of FGDs would provide sufficient information to answer the original study questions. The number of participants in each FGD should ideally be between 6–8 as often recommended,^39^ but due to cancellations the number differed in the end. For each FGD new participants were recruited: FGD 1 with only GPs; FGD 2 with only pulmonologists; FGD 3 with a mix of GPs and pulmonologists. In FGD 1 and 2, GPs and pulmonologists got the chance to discuss issues from the viewpoint of their discipline without being interfered by the other discipline. In FGD 3 these issues could then be combined in an interdisciplinary discussion. The GPs and pulmonologists were sampled in order to cover both rural and urban GP practices, as well as private-based and hospital-based pulmonologists.^15, 38^

All researchers who were responsible for conducting the FGDs participated in a 3-day workshop in order to streamline the methods across countries. All countries used the same interview guide containing topics based on known issues of concern to GPs. The interview guide used in FGD 1 was revised before FGD 2 due to intermediary analysis and this revised version with specific sub questions was used in FGD 2. The pulmonologists were asked to discuss the routines in general practice as they were known to them, but inviting them to be open about their own views and concerns. Between FGD 2 and 3 another revision of the interview guide was made in order to make FGD 3 focus especially on topics that were still unclear but also on solutions to identified problems. All FGDs were transcribed verbatim from audio recording by the local researchers and translated into English by a skilled translator. However, some inconsistencies in English language occurred and we show adjusted quotes in the results section with the original quotes in the attachment. For this analysis, the focus group transcripts were imported into Nvivo 10.

### Data assessment

To address the additional research question for this secondary analysis, we assessed the quality of the available dataset and discussed whether the existing dataset had the potential to inform us about physicians’ engagement in smoking cessation. All transcripts were reviewed by E.E. in order to discover whether they were relevant for this secondary analysis. This was then discussed within the research team (M.B.R., M.S., D.K.) and it was agreed that the dataset as a whole offered rich data on physicians’ engagement in smoking cessation. Most of the data for the secondary analysis arose from questions in all FGDs about difficult COPD patients. FGD 1 and 2 included a question asking, ‘Could you describe specifically challenging or difficult clinical situations with COPD-patients in general? ’ FGD 3 included a question asking, ‘Who are the difficult patients and how do you reach them? What do you think are the key issues/bottlenecks regarding the difficult patients? ’ These questions led to discussions about smoking and smoking cessation in COPD patients. Furthermore, we found valuable information in discussions on challenges when coping with patients with COPD; how to improve care and own views and concerns. Also, sections in which exacerbation management, self-management and guidelines were discussed provided interesting information. Information from FGD 3 contributed the most to this secondary analysis.

### Analysis

The available data were then analysed using an inductive thematic analysis^40^ and we coded the text without initially relating it to the aim of the study. As there were no predefined categories, this enabled the evolution of different categories through the coding process. E.E. formulated codes for all text that was linked to smoking and smoking cessation. Codes were then discussed in detail and sorted into categories and subcategories (E.E., M.B.R.). For example, ‘patients not open to physicians’ smoking cessation advice’ was a subcategory of ‘patients who do not quit’. The codes were then discussed within the research team (M.B.R., M.S., D.K.) and the essential contents of the codes were divided into three candidate themes related to the overall aim. Simultaneously, the final aim of the study was discussed within the team (O.V.S., M.B.R.) and the themes could then be finalised. This process resulted in three themes: (1) ‘physicians’ frustration with COPD patients who smoke’; (2) ‘physicians’ limited knowledge of, and negative beliefs about, smoking cessation treatment’; (3) ‘healthcare organisational factors that influence the use of smoking cessation treatments’. E.E. checked to ensure that the resulting thematic map accurately reflected the meaning of units evident in the dataset as a whole.^40^

### Ethical considerations

Methods were performed in accordance with relevant regulations and guidelines. Relevant ethical approval was obtained from the appropriate bodies in the participating countries; South East Wales Research Ethics Committee Panel (Wales), Medical Ethical Review Committee of University Hospital Maastricht/Maastricht University (The Netherlands), Ethics Committee of Northern State Medical University (Russia). Participants provided written informed consent. Identifiers were removed from the transcriptions locally prior to distribution among the network.

### Data availability

The data that support the findings of this study are available from the corresponding author upon reasonable request and with permission of the lead investigator (HM).

## Electronic supplementary material

Supplementary Info 1 - Interviewguide FGD1 GPs

Supplementary Info 2 - Interviewguide FGD 2 Pulmonologists

Supplementary 3 - Interviewguide FGD 3 GPs &amp; Pulmonologists

Supplementary 4 - Original quotes
