# Supplementary material for: Why do physicians lack engagement with smoking cessation treatment in their COPD patients? A multinational qualitative study
Source: NPJ Prim Care Respir Med. 2017 Jun 23;27:41. doi: 10.1038/s41533-017-0038-6 (PMC5482893; doi:10.1038/s41533-017-0038-6)
Supplement: Supplementary file 1 — Supplementary Info 1 - Interviewguide FGD1 GPs [file 41533_2017_38_MOESM1_ESM.doc]

**Attachment 1: Interviewguide/questioning route for focus group discussions with GPs on assessment and medical treatment of patients with COPD**

Aim: to gain knowledge on current treatment of exacerbations and attitudes to self-treatment and to improve quality of life

*A question that initiated a discussion or provoked statements on smoking or smoking cessation.

**Introduction**:

Present to the participants what the idea of a FGD is and what the topic of the discussion is.

Opening question:

*1) Tell us who you are, where you practice, what are your general thoughts on the management of COPD patients? (all should speak) (5 minutes)

Patient story 1 (told or read aloud):

2) What does the story bring to your mind?

*3) How do you assess and manage exacerbations**?** (10 minutes)

- How should the symptoms of the patients be weighted in the assessment?

- Which examinations and tests would be relevant?

- When would you prescribe systemic (per oral) corticosteroids?

- When would you prescribe antibiotics?

Patient story 2 (told or read aloud)

4) With this we wish to focus on treatment of severe exacerbations more specifically.

When would you consider to hospitalize a patient with COPD exacerbation? (10 minutes)

- If you find it risky to treat the patient in their home, do you see other options than to hospitalize the patient?

(‘Think back’- questions could be used here for prompting)

5) What do you think of self-treatment with antibiotics and oral corticosteroids? (10 minutes)

- Is it common in your setting?

- Do people understand how and why to take the medication?

Patient story 3 (told or read aloud) (optional, depending on the flow of the interview)

6) What could be important areas of self-management for COPD-patients in your opinion?

- What do you think the patients think about self-treatment?

- What role could the patient play in treating their exacerbations?

7) Could you describe in what way you use national and/or international guidelines in your approach to a COPD patient with exacerbations? (15 minutes)

- Do you think the guidelines are specific enough to guide you in your everyday care for COPD patients?

- Do you think the guidelines are validly constructed; good literature search for example?

- Do you know the most important advice from the guideline COPD by heart, or do you need to check papers or internet for that?

*8) Could you describe specifically challenging or difficult clinical situations with COPD-patients in general? (15 minutes)

- in what way are they challenging/difficult?

- how do you deal with them?

- what does your approach depend on?

Summary by moderator or assistant moderator:

9) Do you think this is an adequate summary? (5 minutes)

*10) Of the topics discussed so far, what do you consider to be the most important problem concerning management of COPD patients? (10 minutes)

*11) Of the problems discussed concerning COPD patients and treatment, are there any ideas on areas where clinical practice could be improved? (10 minutes)

- think back on the discussion above of challenges and difficulties

- do not think in terms of solutions necessarily, more on topics.

- try to reflect on everyday problems with an overall view

*12) Are there any topics we missed? That you feel we only treated superficially or that we simply forgot? Anything that you thought you would mention but did not get the chance to? Anything may be important, so feel free! (10 minutes)

Patient stories

1) A 60 year old male patient with moderate COPD has called your practice and asked for medicine, due to increased coughing and shortness of breath the last week. He quit smoking a year ago. You have prescribed anticholinergics for inhalation as maintenance medication. Now he thinks a course of antibiotics might be helpful. He was treated with amoxicillin and prednisolone last winter 9 months ago, and recovered after a few weeks.

2) A 70 year old female patient, still smoking, visits your practice. She was hospitalized due to her COPD one year ago. She uses a combination of inhaled corticosteroids and long acting beta2-agonists, and short acting beta2 agonists on demand. She had a common cold a week ago. Now she has no fever, but breathes heavily and rather fast. She had to sit in her bed last night, and she feels somewhat exhausted. Although you hear wheezes all over her chest, you do not think the obstruction is very severe. You believe her illness is worsened by her anxiety, but consider admitting her to hospital.

3) A 72 year old woman visit you for a follow-up examination. She had a COPD exacerbation three weeks ago, for the second time this winter. She is now in her normal shape. FEV1/FVC ratio is 0.55 and her FEV1 % predicted is 45%. She has reduced her smoking considerably, and smokes only 5 cigarettes a day. She will continue the regular use of a long acting anticholinergic, and is encouraged to use a short-acting beta2 agonist on demand. You consider giving her inhaled corticosteroid in addition. You also consider prescribing courses of oral corticosteroids and antibiotics which she could administer herself if she develops a new exacerbation.
