# Supplementary material for: Why do physicians lack engagement with smoking cessation treatment in their COPD patients? A multinational qualitative study
Source: NPJ Prim Care Respir Med. 2017 Jun 23;27:41. doi: 10.1038/s41533-017-0038-6 (PMC5482893; doi:10.1038/s41533-017-0038-6)
Supplement: Supplementary file 2 — Supplementary Info 2 - Interviewguide FGD 2 Pulmonologists [file 41533_2017_38_MOESM2_ESM.doc]

**Attachment 2: Interviewguide/questioning route for focus group discussions with pulmonologists on assessment and medical treatment of patients with COPD**

Aim: to gain knowledge on current treatment of exacerbations and attitudes to self-treatment and to improve guidelines

*A question that initiated a discussion or provoked statements on smoking or smoking cessation.

**Introduction:**

Present to the participants what the idea of a FGD is and what the topic of the discussion is.

Opening question:

*1) Tell us who you are, where you work and what you think of when talking about COPD patients in general?

Patient story 1 (told or read aloud):

2) What does the story bring to your mind?

*3) What is your opinion about the way primary care manages exacerbations? (10 minutes)

- How should GPs manage exacerbations?

- How should the symptoms of the patients be weighted in the assessment?

- Which examinations and tests would be relevant?

- When should the GP prescribe systemic (per oral) corticosteroids?

- When should the GP prescribe antibiotics?

- How may you describe the treatment approaches to COPD in a hospital context? What takes place? What is being prescribed and advised?

Patient story 2 (told or read aloud)

4) When would you consider hospitalization of a patient with COPD relevant?

- are there any specific criteria?

5) What do you think of self-treatment with antibiotics and oral corticosteroids? (10 minutes)

- Is it common in your setting?

- Do people understand how and why to take the medication?

Patient story 3 (told or read aloud) (optional, depending on the flow of the interview)

6) What do you think the patients think about self-treatment?

- What is important for optimal self-treatment for COPD patients in your opinion?

7) Could you describe in what way you use national and/or international guidelines in your approach to a COPD patient with exacerbations? (15 minutes)

- Are the guidelines relevant for example in a GP setting?

- Do you think the guidelines are specific enough to guide you in your everyday care for COPD patients?

- In what way could they be improved?

- Do you know the most important advice from the guideline COPD by heart, or do you need to check papers or internet for that?

*8) Could you mention specifically challenging or difficult clinical situations with COPD-patients?

- in what way are they challenging/difficult?

- how do you deal with them?

- what does the approach depend on?

Summary by moderator or assistant moderator:

9) Do you think this is an adequate summary?

*10) Of the topics discussed so far, what do you consider to be the most important concerning management of COPD patients?

*11) Of the problems discussed concerning COPD patients and treatment, are there any ideas on areas where clinical practice could be improved?

*12) Are there any topics we missed? That you feel we only treated superficially or that we simply forgot? Anything that you thought you would mention but did not get the chance to? Anything may be important, so feel free! (time enough for this is important)

(Question 13 could be asked at the end if not previously discussed during the FGD – but you also may ask this question whenever you like during the interview if the topic is brought up and you wish to elaborate it)

13) How do you cooperate with general practitioners?

- how can cooperation be improved?

- do you experience delay from general practice or too many admissions?

- how should the division of work be?

Patient stories

1) A GP calls you and asks for advice on a patient: A 60 year old male patient with COPD has called his practice and has asked for some medicine due to increased coughing and shortage of breath over the last week. He quit smoking a year ago and the GP has prescribed anticholinergics for inhalation as a maintenance medication. Now the patient thinks that a course of antibiotics might be helpful. He was treated with amoxicillin and prednisolone last winter, nine months ago and he recovered after a few weeks.

2) A 70 year old female asthma patient, still smoking, visits a GP practice. She was hospitalized due to her asthma/COPD one year ago. She uses a combination of inhaled corticosteroids and long acting beta2-agonists, and short acting beta2-agonists on demand.

She had a common cold a week ago. Now she has no fever, but breathes heavily and rather fast. She had to sit in her bed last night, and she feels somewhat exhausted. Although the GP hears wheezes all over her chest, he does not think the obstruction is very severe. He rather thinks her illness is worsened by her anxiety. Anyway, the GP considers admitting her to hospital.

3) A 72 year old woman visit the GP for a follow-up examination. She had a COPD exacerbation three weeks ago, for the second time this winter. She is now in her normal shape. FEV1/FVC ratio is 0.55 and her FEV1 % predicted is 45%. She has reduced her smoking considerably, and smokes only 5 cigarettes a day. She will continue the regular use of a long acting anticholinergic, and is encouraged to use a short-acting beta2 agonist on demand. The GP considers giving her inhaled corticosteroid in addition. He also considers prescribing courses of oral corticosteroids and antibiotics which she could administer herself if she develops a new exacerbation.
