# Supplementary material for: Why do physicians lack engagement with smoking cessation treatment in their COPD patients? A multinational qualitative study
Source: NPJ Prim Care Respir Med. 2017 Jun 23;27:41. doi: 10.1038/s41533-017-0038-6 (PMC5482893; doi:10.1038/s41533-017-0038-6)
Supplement: Supplementary file 3 — Supplementary 3 - Interviewguide FGD 3 GPs & Pulmonologists [file 41533_2017_38_MOESM3_ESM.doc]

**Attachment 3: Interviewguide/ questioning route for focus group discussions with GPs and pulmonologists on assessment and medical treatment of patients with COPD exacerbations**

Aim: to gain knowledge on current treatment of exacerbations and attitudes to self-treatment and to improve guidelines

*A question that initiated a discussion or provoked statements on smoking or smoking cessation.

**Introduction:**

Present to the participants what the idea of an FGD is and what the topic of the discussion is.

Opening question:

*1) Tell us who you are, where you work and what you think of when talking about COPD patients in general?

2) During our previous discussions in the groups with both pulmonologists and GPs we found that they mostly agreed on how to treat exacerbations, that is when to prescribe antibiotics and steroids and according to which examinations and objective measures. There was however some uncertainty regarding how to diagnose an exacerbation. So our question for you is:

- What do you find difficult when diagnosing a possible exacerbation?

- Is there a diagnostic test you find useful in the situation?

- What are the key issues/bottlenecks in management of exacerbations outside hospital?

- What would be the ideal way to manage exacerbations outside hospital (if you had all the money and resources you could think of)?

- How can you get closer to this ideal and improve the situation?

3) In the previous discussions the physicians tend to agree on severe cases of exacerbation when it comes to hospitalization, whereas there seems to be more confusion about the borderline cases. We are interested in your opinions about who should be admitted apart from severe cases.

- Who should be seen in the hospital and who should not?

- How should the decision be made to admit a patient?

- What should hospitals be used for, only patients in need of intensive care?

- How could admissions of patients with exacerbations be avoided and managed otherwise?

- What do you see as the key issues/bottlenecks/challenges regarding hospitalization?

- What would be the ideal way to manage it (if you had all the money and resources you could think of)?

- How can you get closer to this ideal and improve the situation?

4) When it comes to self-treatment the previous FGDs reveals that the majority thinks it is a feasible and good idea. They do however specify that only certain patients are deemed capable of administering their own medicines. But no one could refer to specific criteria for deciding which patients are capable. Overall we felt the doctors had little experience with self-treatment despite the general positive attitude towards it.

- Why do you think you do not apply self-treatment more?

- What do you need to do this?

- Is it needed in the guidelines?

- What do patients think about self-treatment?

- What do you think are the key issues/bottlenecks regarding self-treatment?

- What would be the ideal way to manage it (if you had all the money and resources you could think of)?

- How can you get closer to this ideal and improve the situation?

5) The next thing we wish to talk about is the collaboration between primary and secondary care.

- How do you collaborate?

(Since there seems to be a consensus in the previous FGDs that there is a need for more collaboration between primary and secondary care, we expect it to be in FGD3 too. If this is not the case, please leave out the first sentence of the following introduction)

There seems to be an agreement that you need to collaborate more, that there is a need to communicate more. This is also what came up in the previous discussions. The physicians mentioned that they liked when there was exchange of experience (at seminars, conferences), and when direct contacts and involvement was made concerning e.g. admission and diagnostics (calling, meetings etc). But they also lacked more of both. It’s easy to say, but apparently difficult to do.

- What do you think are the key issues/bottlenecks regarding collaboration during exacerbations?

- What is the key reason to communicate?

- What can go wrong if you don’t?

- What would be the ideal way to collaborate (if you had all the money and resources you could think of)?

- How can you get closer to this ideal and improve the situation?

*6) The last topic we want you to discuss are the difficult COPD patients in general. In the previous discussions they were described as for example the patients that kept on smoking and the patients who had little to no visible effects of the treatment. Both these factors made it difficult for the doctors to motivate the patients and themselves for further treatment endeavors.

- Who are the difficult patients and how do you reach them?

- What do you think are the key issues/bottlenecks regarding the difficult patients?

- What would be the ideal way to manage them (if you had all the money and resources you could think of)?

- How can you get closer to this ideal and improve the situation?

Summary by moderator or assistant moderator:

7) Do you think this is an adequate summary?

8) Are there any topics we missed? That you feel we only treated superficially or that we simply forgot? Anything that you thought you would mention but did not get the chance to? Anything may be important, so feel free! (time enough for this is important)
