# Supplementary material for: Why do physicians lack engagement with smoking cessation treatment in their COPD patients? A multinational qualitative study
Source: NPJ Prim Care Respir Med. 2017 Jun 23;27:41. doi: 10.1038/s41533-017-0038-6 (PMC5482893; doi:10.1038/s41533-017-0038-6)
Supplement: Supplementary file 4 — Supplementary 4 - Original quotes [file 41533_2017_38_MOESM4_ESM.docx]

Attachment - Original quotes

Theme 1

‘Yes, make them, make them to wish to stop smoking (laughs a bit) is what we should do.’ [FGD 1, Norway, GP]

‘They [COPD patients that smoke] are a very difficult patient group, especially those that are just on the cusp of needing long term oxygen and you’re trying to convince them that you’re not going to give it to them if they don’t stop smoking … it’s very frustrating if they still persist…’ [FGD 3, Wales, Pulmonologist]

‘It’s difficult to treat and handle this group of patients, right, you need to strongly motivate them in order to, if you press them too much you will reject them, if you press them too hard to make them stop smoking you will make them your enemies, that’s the fine line.’ [FGD 1, Norway, GP]

‘I switched to ‘light’ cigarettes’, and ‘Doctor, I’ve dropped out, I do not smoke for three days!’ He smoked for 50 years before’. [FGD 2, Russia, pulmonologists]

‘…it is almost like fighting windmills’ [FGD 3, Poland, pulmonologist]

Theme 2

‘No it is proven if you do not smoke you are better off, if you quit at a young age you are better off, but those people who are in a certain stage of COPD, those data are not convincing…’ [FGD 3, Netherlands, pulmonologist]

‘I would also like to get some new skills about smoking cessation program. We should not only tell patients about quitting smoking, we should offer them something concrete and effective!’ [FGD 2, Russia, pulmonologist]

‘We need resources on creating an atmosphere and educating patients…in order to increase their motivation to quit smoking and increase the success rate…’ [FGD 3, Hong Kong, GP]

‘And the communicative intervention so to say is more successful than, for instance, a nicotine patch.’ [FGD 3, Germany, pulmonologist]

Theme 3

‘It is part of the questionnaire, it is one of the items of the list you need to cover… But it is so time-consuming.’ [FGD 1, Netherlands, GP]

‘It’s not economical but you shouldn’t lose your enthusiasm there.’ [FGD 3, Germany, pulmonologist]

‘I understand that some people have smoking as a disease the same as alcohol dependency, for example.’ [FGD 3, Russia, GP]

‘Because every alcoholic gets detoxicated, but a nicotine patient has to pay for it alone. And this injustice, both are partly recognised as addictions.’ [FGD 3, Germany, GP]
